# Seed-derived peptide lunasin suppressed breast cancer cell growth by regulating inflammatory mediators, aromatase, and estrogen receptors

**DOI:** 10.29219/fnr.v67.8991

**Published:** 2023-01-26

**Authors:** Chia-Chien Hsieh, Chi-Hao Wu, Shih-Han Peng, Chia-Hsin Chang

**Affiliations:** 1 Department of Biochemical Science & Technology, National Taiwan University, Taipei, Taiwan; 2 School of Life Science, Undergraduate and Graduate Programs of Nutrition Science, National Taiwan Normal University, Taipei, Taiwan

**Keywords:** aromatase, breast cancer, lunasin, inflammation, estrogen receptor

## Abstract

**Background:**

Breast cancer is one of the most prevalent cancers in women. Its pathology comprises tumor cells and nearby stromal cells, accompanied by cytokines and stimulated molecules, resulting in a favorable microenvironment for tumor progression. Lunasin is a seed peptide with multiple bioactivities derived from seeds. However, the chemopreventive effect of lunasin on different characteristics of breast cancer has not been fully explored.

**Objective:**

This study aims to explore the chemopreventive mechanisms of lunasin through inflammatory mediators and estrogen-related molecules in breast cancer cells.

**Design:**

Estrogen-dependent MCF-7 and independent MDA-MB-231 breast cancer cells were used. The β-estradiol was used to mimic physiological estrogen. The gene expression, mediator secretion, cell vitality, and apoptosis impacting breast malignancy were explored.

**Results:**

Lunasin did not affect normal MCF-10A cell growth but inhibited breast cancer cell growth, increased interleukin (IL)-6 gene expression and protein production at 24 h, and decreased its secretion at 48 h. In both breast cancer cells, aromatase gene and activity and estrogen receptor (ER)α gene expression were decreased by lunasin treatment, while ERβ gene levels were significantly increased in MDA-MB-231 cells. Moreover, lunasin decreased vascular endothelial growth factor (VEGF) secretion and cell vitality and induced cell apoptosis in both breast cancer cell lines. However, lunasin only decreased leptin receptor (Ob-R) mRNA expression in MCF-7 cells. Additionally, β-estradiol increased MCF-7-cell proliferation but not the proliferation of other cells; in particular, lunasin still inhibited MCF-7-cell growth and cell vitality in the presence of β-estradiol.

**Conclusion:**

Seed peptide lunasin inhibited breast cancer cell growth by regulating inflammatory, angiogenic, and estrogen-related molecules, suggesting that lunasin is a promising chemopreventive agent.

## Popular scientific summary

- Lunasin inhibited growth in breast cancer cells but not in normal cells.
- Lunasin regulated IL-6, COX-2, Ob-R, and VEGF, contributing to chemoprevention.
- Lunasin regulated ERα/β genes and inhibited the aromatase gene and activity in breast cancer cells.
- Lunasin still inhibited cell growth and vitality in MCF-7 cells under β-estradiol.
- Lunasin executes chemoprevention via inflammatory and estrogen-related molecule regulation.

Breast cancer is the most common cancer in women, especially in developed countries. The incidence is increasing globally, thus consuming a tremendous number of medical resources, according to statistical data from the World Health Organization (1). Breast cancer development comprises heterogeneous constituents, including tumor cells and nearby stromal cells, such as fibroblasts, adipocytes, and immune cells, that are involved in maintaining the mammary microenvironment and breast carcinogenesis (2). During breast cancer development, immune mediators, cytokines, and chemokines participate in the pathogenesis of breast cancer and serve as growth-related signals for cancer cells in the microenvironment. The dynamics of these signaling molecules promote or suppress cancer initiation, progression, and therapeutic interventions (3).

Breast cancer is classified as the estrogen receptor (ER)-dependent type, and the ER-independent type refers to the representation of ERs on cells. Approximately 70–80% of breast cancers are estrogen-sensitive and generally treated with conventional therapies, including chemotherapy, radiation, surgery, and hormone analogs. Clinical evidence demonstrates that ER(+) cancer has better therapy and prognosis, while ER(–) cancer is more aggressive and resistant to treatment (4). Over the past decade, the complicated interactions among breast cancer, estrogens, and the immune system have attracted the attention of scientists. Innate and adaptive immunity play a dynamic role during tumor progression or inhibition. Proinflammatory mediators significantly promote tumor formation, and progression in breast cancer has been demonstrated (5). In this microenvironment, inflammatory mediators secreted around the breast tissue have been considered to stimulate aromatase expression and activity, thus triggering estrogen production, especially in obese postmenopausal women (5). Aromatase transfers androstenedione and testosterone to estron and estradiol, displaying the physical form of estrogen *in vivo* (6). Estrogen mediates ERs, including ERα and ERβ, to trigger specific signaling and function in cells. In estrogen-mediated regulation, ERα signaling predominantly executes the simulative immune effect, while ERβ signaling plays an immune suppressive effect (7), indicating that different signaling might affect the course of diseases. Based on this evidence, a close association between the inflammatory response and estrogen biosynthesis and their signaling pathways converge in breast tissue.

Bioactive phytochemicals from food provide valuable health benefits beyond essential nutrition, which efficiently impact specific molecular targets (8). Phytochemicals are non-toxic with a broad range of biological activities and have attracted the interest of scientists as promising therapeutic agents for breast cancer (8). Lunasin is a seed peptide with 43 amino acids found in many legumes and cereals and naturally exists with protease inhibitors, such as Bowman-Birk inhibitor or Kunitz-trypsin inhibitor. Protease inhibitors protect lunasin resistant to digestive enzymes and are intact absorbed and reach tissues and organs (9, 10), possibly through paracellular passive diffusion (11). Lunasin has been demonstrated to have chemopreventive, antioxidant, anti-inflammatory, and immunoregulatory properties, making it a potential peptide against several chronic disorders (9). *In vitro* and *in vivo* studies have demonstrated the chemopreventive properties of lunasin in breast cancer. The possible mechanisms have been explored, including arresting the cell cycle, inducing apoptosis, suppressing histone acetylation, and regulating tumor suppressors and angiogenesis mediators (9, 12–14). Additionally, lunasin exerted anti-inflammatory properties in RAW 264.7 macrophages (15), 3T3-L1 adipocytes (16), and 4T1 triple-negative breast cancer cells (TNBCs) (14). A growing body of evidence has shown that proinflammatory mediators play critical roles closely linked to estrogen production and signaling (17). However, the effect of lunasin on inflammatory mediators and estrogen-related pathways in the breast cancer microenvironment has not been explored.

The main goal of this study was to understand the chemopreventive properties of lunasin in estrogen-dependent and estrogen-independent breast cancers that might benefit cancer therapeutic applications. The possible mechanisms of action involved in inflammatory cytokines and estrogen-related molecules impacting breast malignancy were explored.

## Materials and methods

### Cell culture and treatments

The human breast cancer ER-negative (ER-) MDA-MB-231 cell line and ER-positive (ER+) MCF-7-cell line were purchased from the Bioresource Collection and Research Center (BCRC, Hsinchu, Taiwan), and the human normal mammary epithelial MCF-10A cell line was purchased from the American Type Culture Collection (Manassas, VA, USA). Both cancer cell lines were cultured in Dulbecco’s modified Eagle’s medium (DMEM, Caisson, Smithfield, UT, USA) containing 10% fetal bovine serum (FBS, Genedirex, Las Vegas, NV, USA) with 1% antibiotic antimycotic solution (Caisson). Mammary epithelial MCF-10A cells were cultured in DMEM/nutrient mixture F-12 (DMEM/F12, Gibco, Grand Island, NY, USA) with 5% horse serum (Gibco), 20 ng/mL recombinant human EGF (Peprotech, Rocky Hill, NJ, USA), 0.5 μg/mL hydrocortisone (Sigma, St. Louis, MO, USA), 10 μg/mL insulin (Sigma), and 1% antibiotic solution. All cells were maintained in a 37°C incubator with a humidified atmosphere containing 5% CO_2_. Lunasin was chemically synthesized with a purity higher than 95% (Kaijie Peptide Company, Chengdu, China). A stock solution of synthetic lunasin was prepared with sterile distilled water and stored at −20°C.

### Cell viability assay

MCF-10A cells were seeded at 1 × 10^4^ cells/well, and MDA-MB-231 and MCF-7 cells were seeded at 5 × 10^3^ cells/well in 96-well plates (Becton Dickinson, Franklin Lakes, NJ, USA) and treated with 5, 10, 25, 50, 100, or 200 μM lunasin in complete media concurrently for 24, 48, and 72 h. After culture, the cells were incubated in 50 mL of 0.5 μg/mL 3-(4,5-dimethylthiazol-2-yl)-2,5-diphenyltetrazolium bromide (MTT, Sigma) for 3 h. Supernatants were removed, and formazan crystals were solubilized by dimethyl sulfoxide (DMSO, Sigma). The absorbance of the microplate was measured at 540 nm using a spectrophotometric reader (BioTek, Winooski, VT, USA). The cell viability of each sample was calculated as the percentage of control, followed by the formula (sample absorbance – blank absorbance)/(control absorbance – blank absorbance) × 100.

### Gene expression profiling using quantitative PCR

Cells were plated in a 6-well plate overnight and then treated with lunasin for another 24 h. Cells were harvested, and total RNA was extracted by Quick-RNA^TM^ MiniPrep (Zymo Research, CA, USA). Appropriate RNA from each sample was used to perform reverse transcription using an iScript^TM^ cDNA synthesis kit (Bio-Rad Laboratories Inc., CA, USA), according to the manufacturer’s instructions. The cDNA samples were incubated with each target gene, including glyceraldehyde-3-phosphate dehydrogenase (GAPDH), ERα, ERβ, aromatase, cyclooxygenase (COX)-2, hypoxia-inducible factor (HIF)-1α, leptin receptor (Ob-R), and interleukin (IL)-6 Taqman probe (Applied Biosystems, Waltham, MA, USA) with 10 μL iQ Supermix (Bio-Rad, Hercules, CA, USA). The following probes were used: GAPDH (Hs02786624_g1), COX-2 (Hs00153133_m1), HIF-1α (Hs00153153_m1), Ob-R (Hs00174497_m1), IL-6 (Hs00174131_m1) ERα (Hs00174860_m1), ERβ (Hs01100353_m1), and aromatase (Hs00903411_m1). Then, mixtures were processed for quantitative real-time polymerase chain reaction, and genome expression was determined by a StepOnePlus^TM^ real-time system (Applied Biosystems). Specific genes were quantified based on the cycle threshold (Ct) number indicating the fractional cycle number, where the fluorescent reporter signal reached the detected threshold. The normalized ΔCt value of each transcript was calculated using GAPDH as the endogenous control gene, following (target gene Ct) – (internal control Ct) = ΔCt. Gene expression of treatment presented as a fold change was using 2^-ΔΔCt^ for a gene relative to that gene expressed in the control sample.

### Cytokine production

Cells were plated at 2 × 10^5^/well in a 12-well plate overnight and then treated with 0, 5, and 50 μM lunasin for 24 or 48 h. Supernatants of cell culture were harvested, and cytokine production, including vascular endothelial growth factor (VEGF) (R&D, Minneapolis, MN, USA), IL-6 (BioLegend, San Diego, CA, USA), and IL-1β (BioLegend), was measured by enzyme-linked immunosorbent assay (ELISA), according to the manufacturer’s protocol. Briefly, plates were coated with capture antibodies. After overnight incubation, the plates were washed, blocked, and washed again. Then, standard solutions or supernatants were added and incubated for 2 h. After washing, plates were added and incubated with detection antibodies, horseradish peroxidase-conjugated streptavidin, and, finally, the substrate solution. The absorbance of the plate was measured using a spectrophotometer (Molecular Devices, Sunnyvale, CA, USA). Cytokine production was calculated with standard cytokine curves. The unit of concentration was pg/mL, and the data were present as followed, (sample level)/(control level) × 100.

### Prostaglandin E_2_ (PGE_2_) production

Cells were plated at 2 × 10^5^/well in a 12-well plate overnight and then treated with 0, 5, and 50 mM lunasin for 24 or 48 h. Supernatants of culture were harvested, and PGE_2_ production was analyzed. The PGE_2_ level was measured using the prostaglandin E_2_ ELISA Kit (Caymen Chemical, Ann Arbor, Michigan, USA), according to the manufacturer’s instructions. Briefly, standard or samples with PGE_2_ AchE tracer and monoclonal antibody were mixed, added to wells, and incubated for 18 h. After washing, Ellman’s reagent was added to obtain a color reaction. Absorbance was measured using a spectrophotometer (Molecular Devices, Sunnyvale, CA, USA), and the concentration was calculated according to the standard curve. The unit of concentration was pg/mL, and the data were present as followed, (sample level)/(control level) × 100.

### Cell aromatase assay

Aromatase plays a critical role in steroidogenesis, catalyzing androgenic hormones into estrogens. MDA-MB-231 and MCF-7 breast cancer cells were seeded at 5 × 10^5^/well in a 6-well plate overnight and then treated with 0, 5, and 50 μM lunasin for 48 h. Cells were harvested, cellular protein was extracted, and aromatase activity was analyzed using an aromatase (CYP19A) activity assay kit (BioVision), following the manufacturer’s manual. Briefly, cells were collected and washed, aromatase assay buffer was added to cell pellets, cells were sonicated, and cell lysates were assayed. The cell lysate was added to 2 μL nicotinamide adenine dinucleotide phosphate (NADPH) generating system, absent or present aromatase inhibitor letrozole, and then adjusted to the final volume with assay buffer for 20 min at 37°C. Then, an aromatase substrate/β-NADP^+^ mixture was added to each well. Aromatase-specific activity was measured as a fluorescent metabolite in the visible range (Ex/Em = 488/527 nm) every 5 min and calculated by continually parallel reactions in the presence and absence of the inhibitor and subtracting the residual activity detected with the inhibitor present.

### Cell viability in the β-estradiol model

MCF-10A cells were seeded at 1 × 10^4^ cells/well, and MDA-MB-231 and MCF-7 cells were seeded at 5 × 10^3^ cells/well in 96-well plates (Becton Dickinson) for attachment and then treated with serious doses at 1, 5, 20, 50, and 100 nM β-estradiol (E2, Sigma) in complete media concurrently for 24 and 48 h. After culture, the cells were incubated with 0.5 mg/mL MTT for 3 h. Supernatants were removed, and formazan crystals were solubilized by adding DMSO. The absorbance was measured at 540 nm using a spectrophotometric reader (BioTek). After the E2 condition has been set, cells were seeded, treated with 0, 5, and 50 μM lunasin and 20 nM E2 at the same time for 24 and 48 h, and then cell viability was analyzed. The cell viability of every sample was calculated as a percentage of the control.

### Cell vitality

The vitality assay determines the level of reduced thiols, such as glutathione (GSH). GSH oxidation status detects the thiol-disulfide status in the cells by thiol-disulfide interchange reactions. The free thiols on a single cell were stained by VB-48™ fluorescence, thus determining healthy cell status. MDA-MB-231 and MCF-7 cells were seeded at 1.2 × 10^5^ cells/well in 24-well plates. After attachment, both cells were changed to serum-free medium, and MCF-7 cells were cultured in medium without phone red for 24 h. Both types of cells were treated with lunasin for 48 h. At the same time, MCF-7 cells were cultured in the presence or absence of 20 nM β-estradiol, and then the cells were harvested. A cell suspension was added to 1 μL VB-48^TM^ PI-AO (ChemoMetec, Allerød, Denmark), and a 10 μL mixture was taken for analysis using the NucleoCounter® NC-3000™ (ChemoMetec). The data were used to calculate the fluorescence intensity in each population, which identifies VB-48™-positive cells as healthy cells and propidium iodide (PI)-negative cells with low VB-48™ as low vitality, according to the manual.

### Cell apoptosis

MDA-MB-231 and MCF-7 cells were seeded in 24-well plates. After attachment, both cells were changed to serum-free medium, and MCF-7 cells in the β-estradiol condition were cultured in phenol red-free medium for 24 h. Then, the cells were treated with lunasin for 48 h and harvested. The cell suspension was added to the CF™488A Annexin V-CF488A conjugate antibody (ChemoMetec) in Annexin binding buffer (ChemoMetec) and mixed well according to the manufacturer’s instructions. After washing, the cells were stained with PI (Sigma) in Annexin binding buffer, and the data were analyzed and acquired by a NucleoCounter® NC-3000™ (ChemoMetec). The percentage of total apoptotic events showed the sum of the population in the early (Annexin V-positive) and the late (Annexin-V-positive, PI-positive) stages.

### Statistical analysis

All data are presented as the mean ± standard error of the mean (SEM), and each assay was processed in at least three independent experiments. Statistically significant differences between the control and treatments were analyzed by independent-sample *t-*test using IBM Statistical Product and Service Solutions (SPSS version 23, IBM, New York, USA). A *P-*value of less than 0.05 was considered statistically significant.

## Results

### Lunasin inhibited the viability of breast cancer cells

**Fig. 1:**
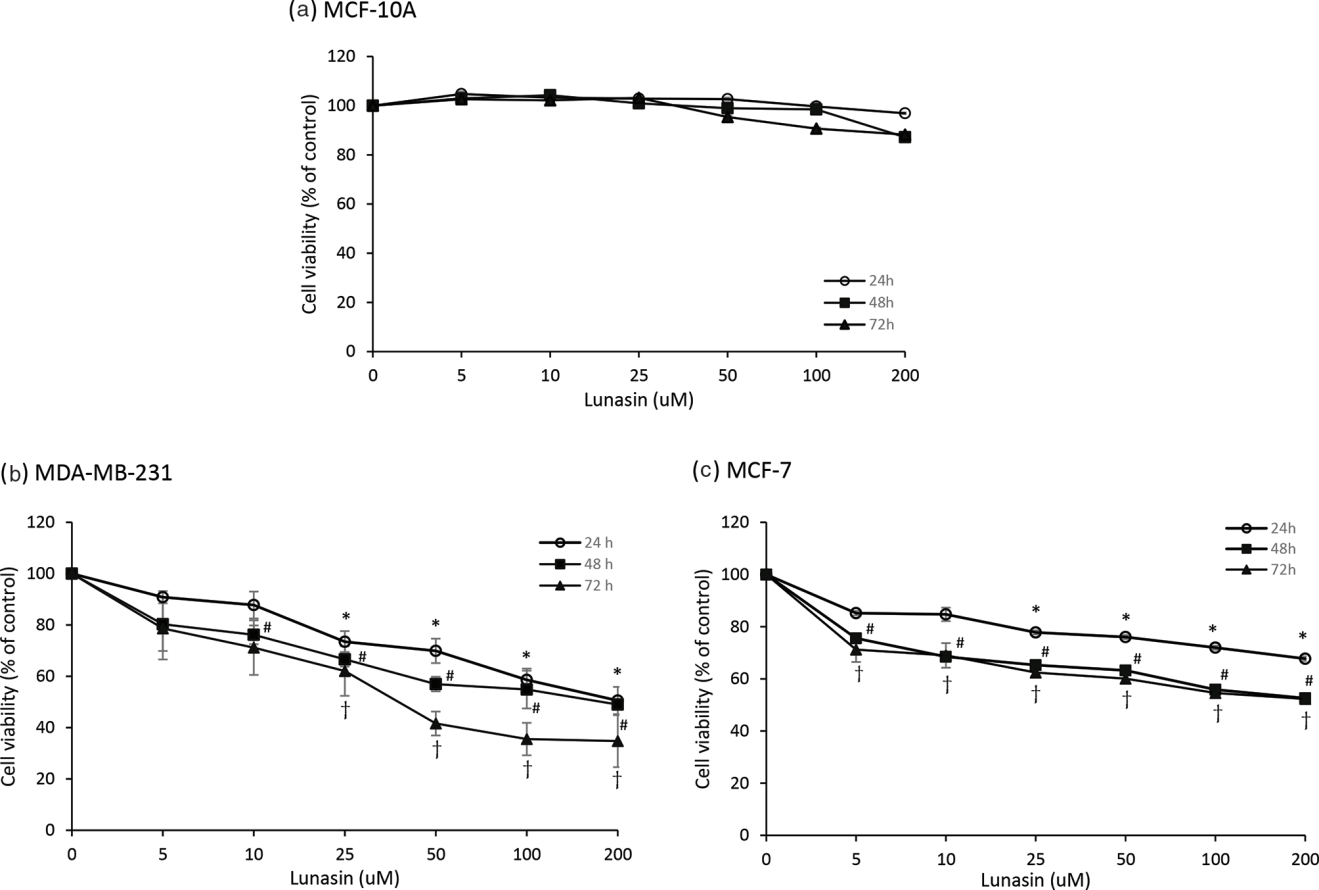
Lunasin inhibited the viability of breast cancer cells. (a) MCF-10A cells, (b) MDA-MB-231 cells, and (c) MCF-7 cells were treated with various doses of lunasin for 24, 48, and 72 h, and the cell viability was determined by MTT assay. The values of the data are presented as the mean ± SEM from at least three independent experiments. Statistical analysis was performed by independent sample *t*-test; significant differences are displayed as follows: *P < 0.05 for 24 h; ^#^*P* < 0.05 for 48 h; ^†^*P* < 0.05 for 72 h, which indicates lunasin treatment compared to the control group.

We examined the effects of lunasin on cell viability by the MTT assay. MCF-10A normal cells and MDA-MB-231 and MCF-7 breast cancer cells were treated with serial doses of lunasin for 24, 48, and 72 h (Fig. 1a–c). Lunasin treatments did not affect the growth of MCF-10A normal cells (Fig. 1a). In MDA-MB-231 cells, lunasin inhibited cell viability at doses higher than 25 μM at 24, 48, and 72 h (*P* < 0.05) (Fig. 1b). In MCF-7 cells, lunasin inhibited cell viability at doses higher than 25 μM at 24 h and 5 μM at 48 h and 72 h (*P* < 0.05) (Fig. 1c). The cell numbers depended on the dosage for lunasin treatment and were calculated with IC_50_ values of 153 μM and 232 μM at 48 h in MDA-MD-231 and MCF-7 cells, respectively. Lunasin treatment did not affect normal cell growth but inhibited the growth of both breast cancer cell lines in a dose-dependent manner.

### Lunasin regulates gene expression and secretion of carcinogenic mediators

**Fig. 2:**
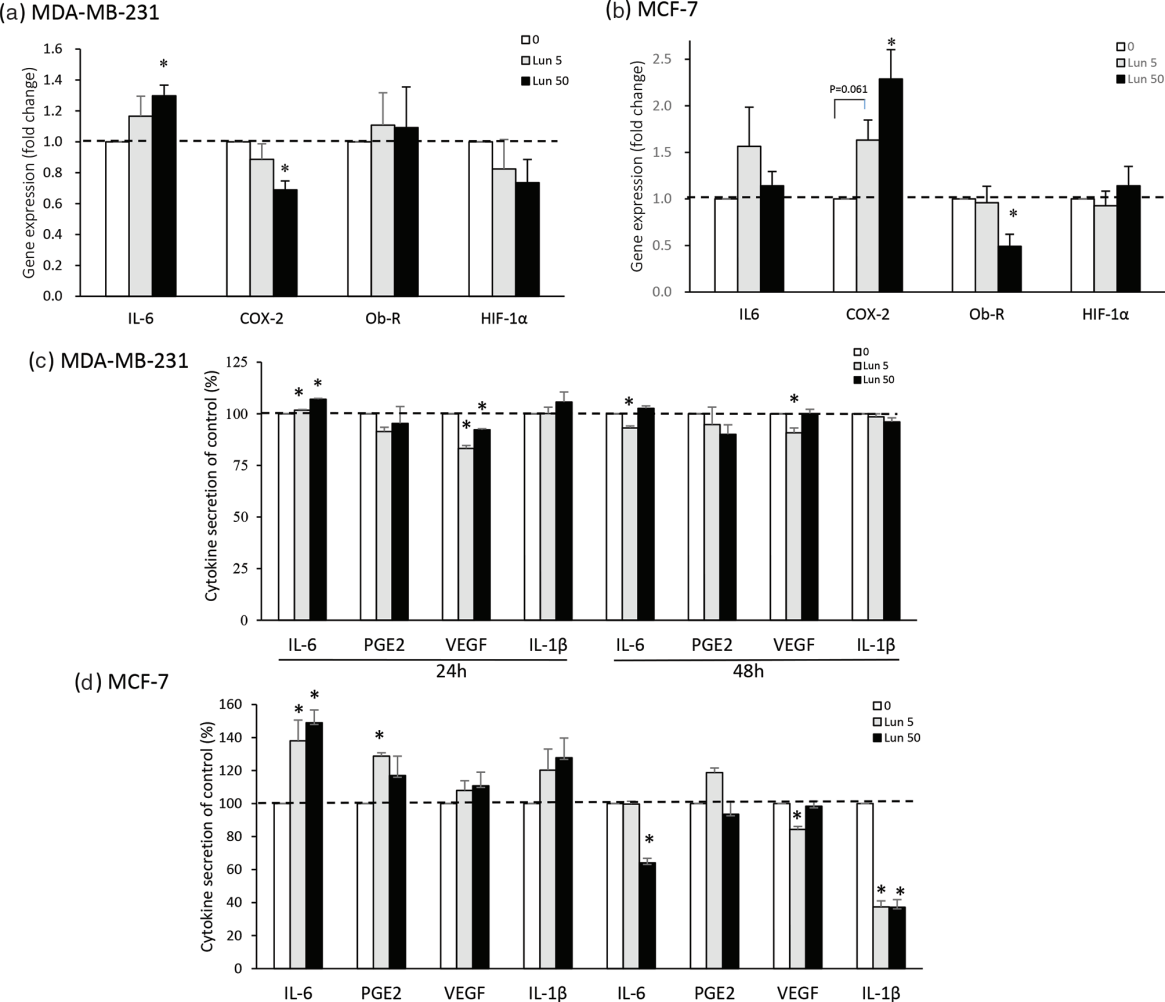
The effects of lunasin on carcinogenic-related gene expression and protein secretion in breast cancer cells. Carcinogenic-related gene expression is shown in (a) MDA-MB-231 cells and (b) MCF-7 cells treated with lunasin for 24 h, and the gene expression was measured by qPCR. The specific gene expression is shown as the fold change compared with the control group. Cytokine production is shown in (c) MDA-MB-231 cells and (d) MCF-7 cells treated with lunasin for 24 and 48 h, and the supernatants were analyzed by ELISA. The value of data is presented as mean ± SEM, and statistical analysis was done by independent sample *t*-test; significant differences of control displayed in *P < 0.05.

Evidence has demonstrated that overexpressed inflammatory mediators might affect breast cancer progression and prognosis (3, 5). Therefore, the gene expression of these carcinogenic mediators in both breast cancer cell lines after lunasin treatments for 24 h was analyzed by quantitative polymerase chain reaction (qPCR), as shown in Fig. 2. To determine the relative expression of each gene, the cycle numbers of qPCR analysis are listed in Supplementary 1. In MDA-MB-231 cells treated with 50 μM lunasin, COX-2 mRNA levels were reduced by 0.31-fold, but IL-6 levels were increased 0.30-fold (Fig. 2a). In MCF-7 cells treated with 50 μM lunasin, the COX-2 mRNA level was increased by 1.29-fold, but the Ob-R level was inhibited 0.51-fold (Fig. 2b). However, lunasin treatments did not affect HIF-1α mRNA expression in either cell line.

The secretion of inflammatory mediators in the culture supernatants of breast cancer cells was determined by ELISA. The quantity of cytokine secretion is shown in Supplementary 2, and the percentage was calculated as the treatment compared to the control group, as shown in Fig. 2c and d. The secretion of VEGF in MDA-MB-231 cells was 3-fold higher than that in MCF-7 cells. In MDA-MB-231 cells, lunasin first increased IL-6 secretion at 24 h and then decreased IL-6 and VEGF secretion at 48 h (Fig. 2c). In MCF-7 cells, lunasin increased IL-6 and PGE2 productions at 24 h but decreased IL-6, IL-1β, and VEGF levels at 48 h (Fig. 2d). Lunasin treatment of both cancer cell lines increased IL-6 gene and protein expression at 24 h and then decreased its protein production at 48 h while increasing COX-2 gene and PGE2 level at 24 h in MCF-7 cells but not in MDA-MB-231 cells. Lunasin regulated inflammatory mediators and angiogenic VEGF secretion in estrogen-dependent and estrogen-independent breast cancer cells in different ways.

### Lunasin regulates estrogen receptor gene expression and aromatase activity

**Fig. 3:**
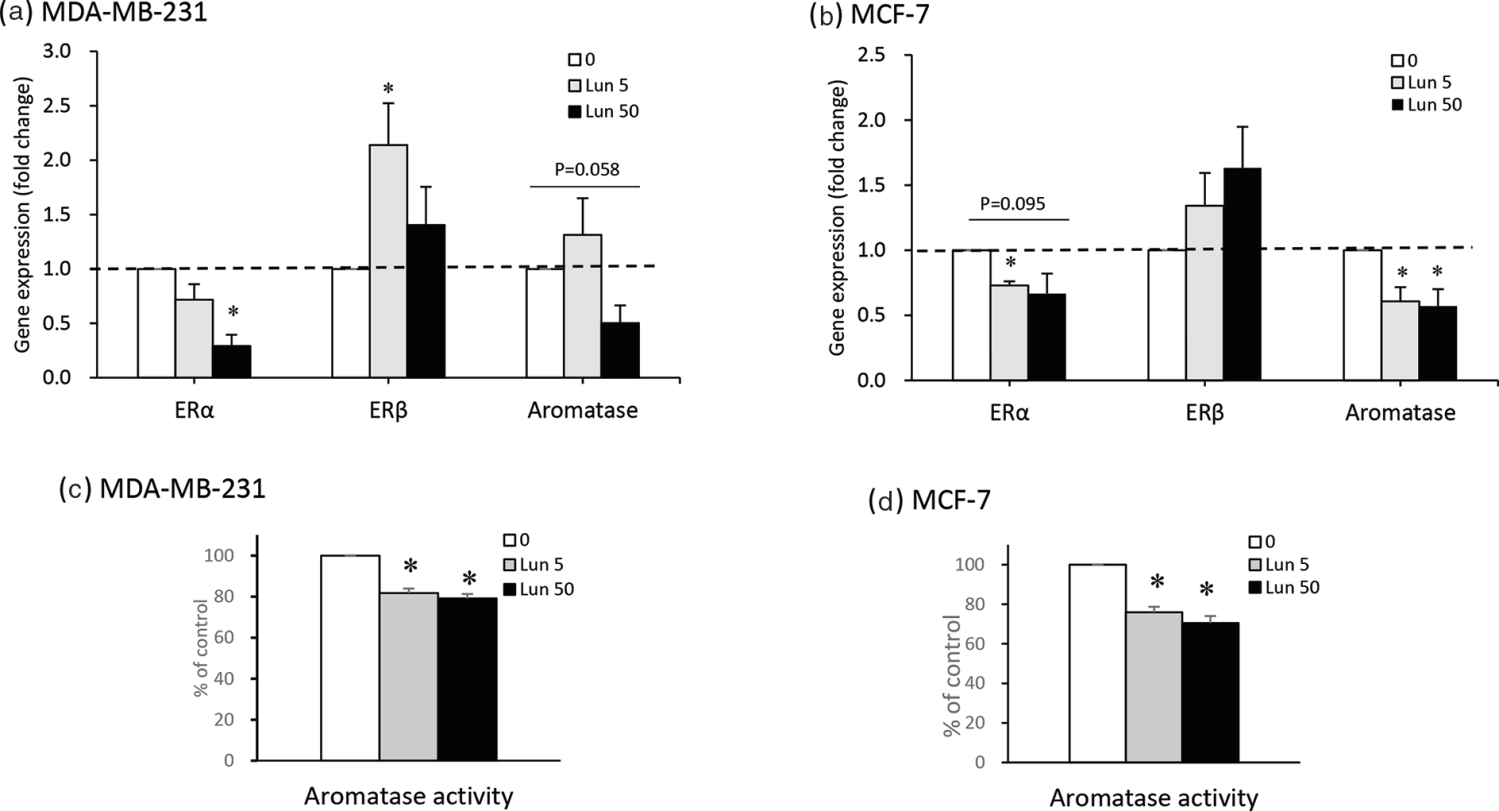
The effects of lunasin on estrogen receptor and aromatase gene expression and aromatase activity in breast cancer cells. Gene expression is shown in (a) MDA-MB-231 cells and (b) MCF-7 cells treated with lunasin for 24 h, and the gene expression was measured by qPCR. The specific gene expression is shown as the fold change compared with the control group. The aromatase activity was determined in (c) MDA-MB-231 cells and (d) MCF-7 cells treated with lunasin for 48 h. The value of data is presented as mean ± SEM, and statistical analysis was done by independent sample *t*-test; significant differences of control displayed in *P < 0.05.

Regarding the estrogenic pathway, ERα, ERβ, and aromatase gene expression were analyzed by qPCR in both cancer cell lines treated with lunasin (Fig. 3a, b). Lunasin treatment decreased ERα gene expression in both breast cancer cell lines, but the ERβ level was significantly increased in MDA-MB-231 cells. The aromatase gene expression was decreased in MCF-7 cells treated with 5 and 50 μM lunasin (*P* < 0.05) and MDA-MB-231 cells treated with 50 μM lunasin (*P* = 0.058) (Fig. 3a, b). The aromatase enzyme activity was analyzed in both types of cells to confirm the biofunction of aromatase. The enzyme activity data showed a decrease in MDA-MB-231 and MCF-7 cells by 50 μM lunasin at 21 and 30%, respectively (*P* < 0.05), in a dose-dependent manner (Fig. 3c, d).

### Lunasin inhibited the viability of breast cancer cells under β-estradiol conditions

**Fig. 4:**
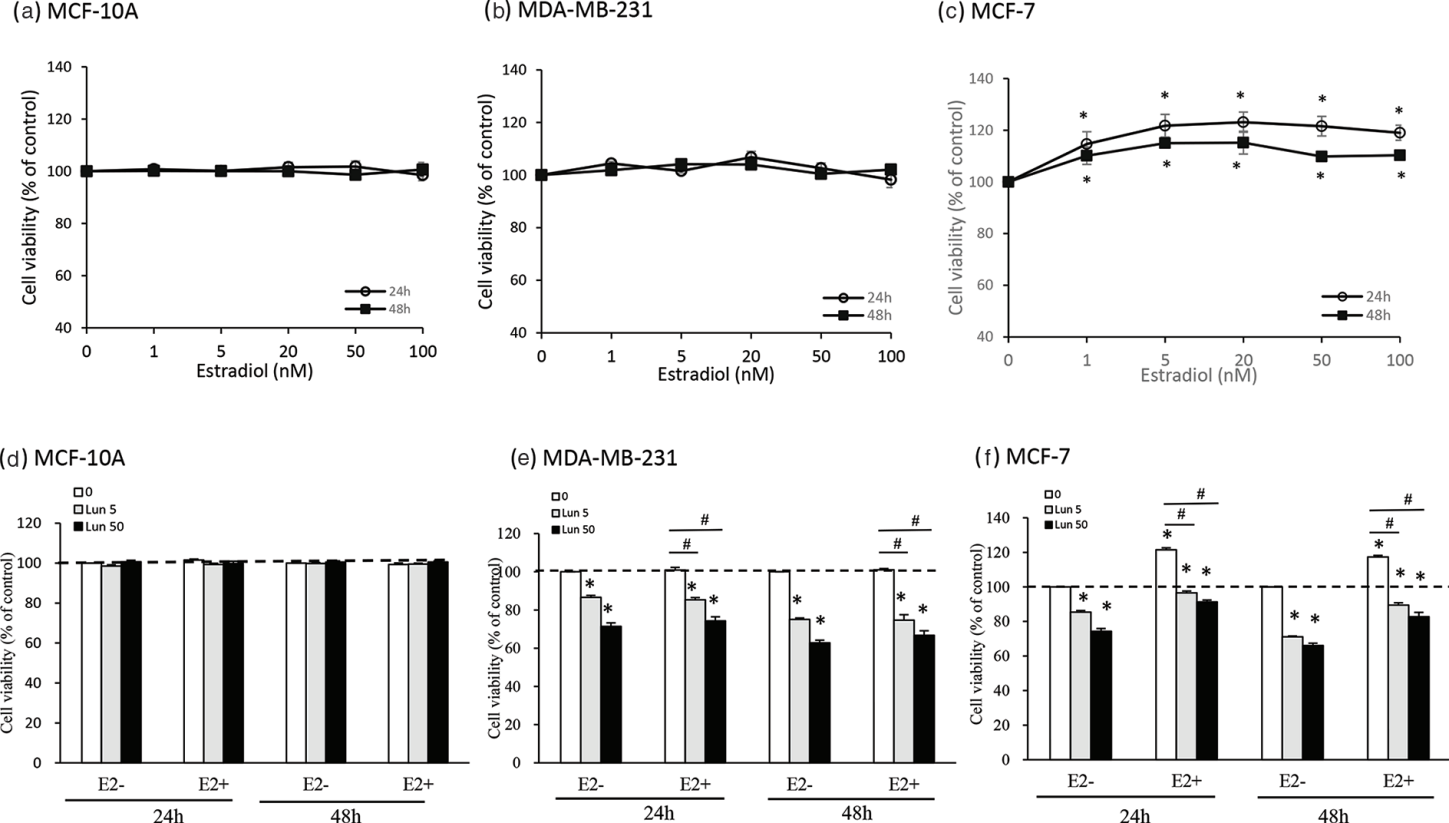
The effects of β-estradiol and lunasin on cell viability in breast cancer cells. (a) MCF-10A cells, (b) MDA-MB-231 cells, and (c) MCF-7 cells were treated with various doses of β-estradiol (E2) for 24 and 48 h. Lunasin affected the viability of (d) MCF-10A cells, (e) MDA-MB-231 cells, and (f) MCF-7 cells treated with 20 nM E2 for 24 and 48 h, and the cell viability was determined by the MTT assay. The data are presented as the mean ± SEM from three independent experiments. Statistical analysis was performed by independent sample *t*-test; significant difference displayed in symbols: *P < 0.05, lunasin treatment versus control without E2; ^#^*P* < 0.05, lunasin treatment versus control with E2.

To investigate whether estrogen affects cell proliferation dependent on ERs expressed on cells, cell viability was determined using the MTT assay after cells were treated with β-estradiol. Normal MCF-10A cells, breast cancer MDA-MB-231 cells, and MCF-7 cells were treated with serial doses of β-estradiol for 24 or 48 h. β-Estradiol treatment did not affect the viability of MCF-10A cells or estrogen-independent MDA-MB-231 cells at 24 and 48 h (Fig. 4a, b). However, β-estradiol significantly increased MCF-7-cell proliferation in a dose-dependent manner at concentrations ranging from 1 to 100 nM at 24 or 48 h (*P* < 0.05) (Fig. 4c). This result indicated that β-estradiol treatment did not affect estrogen-independent breast cancer cell growth but significantly stimulated estrogen-dependent breast cancer cells. The dosage of 20 nM estradiol was used as the estrogen physiological present in the following experiment, referring to the saturated effect of cell proliferation shown at this dosage.

Breast cancer cells treated with lunasin were absent or present with β-estradiol, and cell viability was analyzed to confirm lunasin’s suppressive action under the β-estradiol condition. Lunasin did not affect normal MCF-10A cell growth (Fig. 4d), and these data were consistent with Fig. 1a. In MDA-MB-231 cells, regardless of whether β-estradiol was absent or present, cell growth was not changed, while the suppression of cell viability by lunasin was the same (Fig. 4e). In MCF-7 cells, β-estradiol treatment significantly enhanced cell proliferation, and lunasin still inhibited cell growth regardless of whether β-estradiol was absent or present (*P* < 0.05) (Fig. 4f).

### Cell vitality of breast cancer cells treated with lunasin

**Fig. 5:**
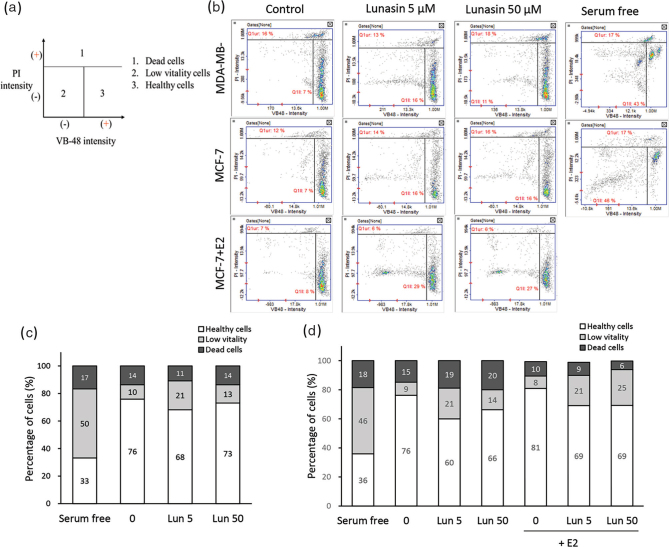
Cell vitality of breast cancer cells treated with lunasin. MDA-MB-231 and MCF-7 cells were treated with lunasin for 48 h, MCF-7 cells were treated with or without 20 nM E2 at the same time, and the cells were collected. The cell vitality was analyzed: (a) definition of histogram plots and (b) fluorescent intensity plots, (c) the proportion of MDA-MB-231 cells, and (d) the proportion of MCF-7 cells. This figure represents the analytic pattern by the NC-3000, and the experiment was performed in at least three independent experiments.

The vitality assay determines the level of reduced thiols, such as GSH, thus determining healthy cell status. It has been demonstrated that the cell vitality of thiol staining can be used to evaluate cellular health, oxidative stress, and indirect apoptosis (18). The definition of dead cells with a high level of PI staining is zone 1, the definition of low vitality cells with low thiols and PI staining is zone 2, and the definition of healthy cells with high thiols is zone 3 (Fig. 5a). The histogram plot of the assay is shown in Fig. 5b, and the data were quantified as shown in Fig. 5c and d, and Table 1. Both cell lines were cultured in serum-free medium as an internal control, and cells had a higher population of low vitality cells and a lower population of healthy cells. In MDA-MB-231 cells, 5 μM lunasin increased the number of low vitality cells (*P* < 0.05) and decreased the number of healthy cells (*P* = 0.066). In MCF-7 cells, 5 and 50 μM lunasin increased low vitality cells (*P* < 0.05) and decreased healthy cells (*P* ≤ 0.05), as well as in β-estradiol condition, lunasin also executed the same effect as increased the low vitality cells and decreased the healthy cells (Table 1).

**Table 1:** Cell vitality of breast cancer cells treated with lunasin^a,b^

	Dead cells	Low vitality	Healthy cells
E2 (-)	MDA-MB-231 cells (mean ± SEM)		
Serum free	16.6 ± 1.18	50.2 ± 5.52	33.2 ± 5.99
0	13.6 ± 1.89	10.5 ± 1.03	75.9 ± 1.87
Lun 5 μM	10.8 ± 1.70	21.0 ± 1.98*	68.2 ± 3.12^†^
Lun 50 μM	13.7 ± 2.39	13.3 ± 1.26	73.1 ± 2.95
E2 (-)	MCF-7 cells (mean ± SEM)		
Serum free	18.5 ± 2.61	45.6 ± 2.57	35.9 ± 2.21
0	14.8 ± 2.06	9.02 ± 0.71	76.2 ± 2.50
Lun 5 μM	18.8 ± 2.68	21.2 ± 2.91*	60.0 ± 5.33*
Lun 50 μM	20.0 ± 2.75	13.7 ± 1.05*	66.3 ± 3.47*
E2 (+)	MCF-7 cells (mean ± SEM)		
0	9.90 ± 1.48	8.47 ± 0.92	80.96 ± 1.98
Lun 5 μM	8.96 ± 1.51	20.78 ± 4.85^†^	69.13 ± 3.69*
Lun 50 μM	5.92 ± 1.97	24.62 ± 1.47*	69.23 ± 2.39*


a MDA-MB-231 cells and MCF-7 cells were absent or present with β-estradiol (E2), and cells were treated with lunasin for 48 h. Cells were collected, and cell vitality was analyzed by NC-3000.

b The value of data is presented at least three independent experiments as mean ± SEM, and statistical analysis was done by independent sample *t*-test; significant differences of control displayed in

† 0.1 > *P* > 0.05 and

* *P* < 0.05.

### Cell apoptosis of breast cancer cells treated with lunasin

**Fig. 6:**
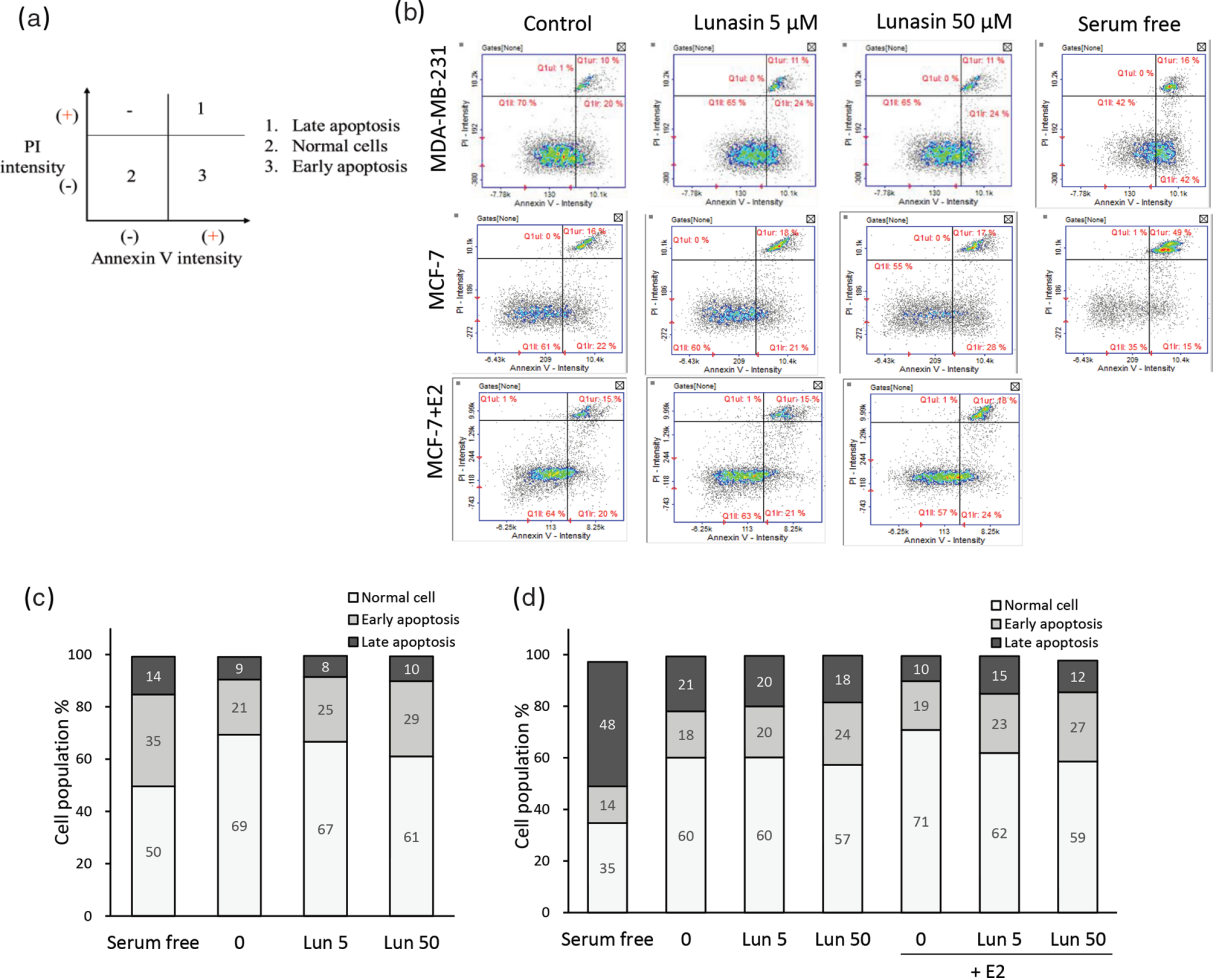
Cell apoptosis of breast cancer cells treated with lunasin. MDA-MB-231 and MCF-7 cells were treated with lunasin for 48 h, MCF-7 cells were treated with or without 20 nM E2 at the same time, and the cells were collected. Cell apoptosis was analyzed: (a) the definition of histogram plots and (b) fluorescent intensity plots, (c) the proportion of MDA-MB-231 cells, and (d) the proportion of MCF-7 cells. The figure represents the analytic pattern by the NC-3000, and the experiment was performed in at least three independent experiments.

To confirm the cell apoptotic stages, cells were treated with lunasin and β-estradiol and then stained with annexin V and PI (Fig. 6a, b), and their quantity is shown in Fig. 6c and d, and Table 2. MDA-MB-231 cells and MCF-7 cells were cultured in serum-free medium as an internal control, and both cell lines had approximately 50 and 62% of the total apoptosis (Annexin-V+ and Annexin-V+/PI+). In MDA-MB-231 cells, 50 μM lunasin tended to increase the early stage (Annexin-V+) of apoptosis (*P* = 0.078) and increase the total stage of apoptosis (*P* < 0.05). In MCF-7 cells, lunasin increased the early stage of apoptosis in a dose-dependent manner (*P* < 0.05), and 50 μM lunasin tended to increase total apoptosis (*P* = 0.067). Under the β-estradiol condition, treatment with lunasin did not significantly induce MCF-7 breast cancer cell apoptosis (Table 2).

**Table 2:** Cell apoptosis of breast cancer cells treated with lunasin^a,b^

	Early apoptosis	Late apoptosis	Total apoptosis
E2 (-)	MDA-MB-231 cells (mean ± SEM)		
Serum free	35.1 ± 7.62	14.4 ± 2.02	49.5 ± 6.06
0	21.1 ± 2.90	8.61 ± 1.54	29.7 ± 1.41
Lun 5 μM	24.8 ± 2.26	7.98 ± 1.53	32.8 ± 1.37
Lun 50 μM	28.8 ± 2.17^†^	9.51 ± 1.47	38.3 ± 1.25*
E2 (-)	MCF-7 cells (mean ± SEM)		
Serum free	14.2 ± 1.27	48.2 ± 1.53	62.4 ± 2.16
0	18.0 ± 1.73	21.3 ± 2.11	39.3 ± 0.73
Lun 5 μM	19.8 ± 1.42	19.5 ± 1.61	39.3 ± 2.05
Lun 50 μM	24.2 ± 1.65*	18.1 ± 0.66	42.4 ± 1.19^†^
E2 (+)	MCF-7 cells (mean ± SEM)		
0	18.9 ± 4.61	9.71 ± 2.77	28.6 ± 3.51
Lun 5 μM	23.0 ± 6.85	14.6 ± 0.59	37.5 ± 7.24
Lun 50 μM	26.9 ± 8.11	12.3 ± 2.05	39.2 ± 7.99


a MDA-MB-231 and MCF-7 cells were absent or present with β-estradiol (E2), and cells were treated with lunasin for 48 h. Cells were collected, and apoptosis was analyzed by NC-3000.

b The value of data is presented at least three independent experiments as mean ± SEM, and statistical analysis was done by independent sample *t*-test; significant differences of control displayed in

† 0.1 > *P* > 0.05 and

* *P* < 0.05.

## Discussion

Cancer is the most challenging medical issue and consumes vast amounts of resources worldwide. The general therapeutic strategies for breast cancer are surgery, chemotherapy, radiotherapy, and targeted approaches such as anti-estrogens. However, these therapies have many side effects, and targeted treatment easily develops drug resistance, particularly in ER+ breast cancer (8). Bioactive phytochemicals derived from seeds provide valuable health benefits beyond essential nutrition. Lunasin, a natural peptide with multiple bioactivities, was found in comment legumes and cereals (9).

**Fig. 7:**
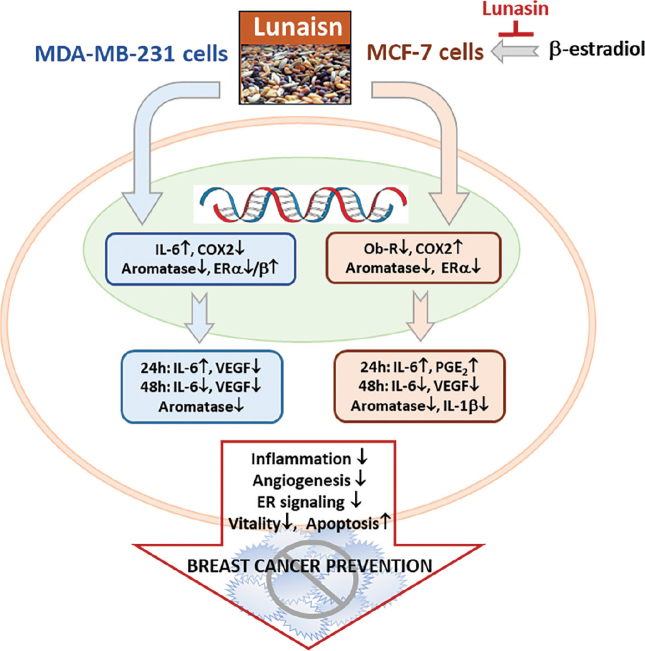
Proposed scheme of the possible mechanism by which lunasin suppresses the growth of breast cancer cells. Lunasin regulated inflammatory mediators, estrogen receptors gene expression, and aromatase activity, resulting in apoptosis induction and decreased cell viability and contributing to suppressing breast cancer progression. Abbreviations: COX-2, Cyclooxygenase-2; ER, estrogen receptor; IL, interleukin; PGE_2_, prostaglandin E_2_; VEGF, vascular endothelial growth factor.

This is the first study that demonstrated that lunasin-regulated inflammatory mediators and ERs expression, inhibited aromatase activity, and consequently induced apoptosis and decreased cell growth in MDA-MB-231 and MCF-7 breast cancer cells but did not affect MCF-10A normal cells. Additionally, the β-estradiol condition was used to mimic physiological estrogen present. It has been shown to increase MCF-7-cell proliferation, and lunasin still executes its suppressive properties in cell vitality and growth. Based on these data, lunasin is a promising natural agent for chemoprevention in breast cancer cells. This suppressive effect was especially sustained under the β-estradiol condition in ER+ breast cancer cells. The proposed scheme of the possible mechanism by which lunasin suppresses breast cancer cell growth is shown in Fig. 7.

Recently, Kawaguchi and coworkers revealed that the expression of several specific inflammatory cytokines in serum was different between breast cancer patients and healthy volunteers (19). Therefore, cytokines could be considered effective strategies for breast cancer therapy. In MCF-7 and MDA-MB 231 cells, lunasin treatment moderately increased IL-6 secretion and subsequently decreased cell numbers, referred to inflammatory-related mediators were cytotoxic. However, evidence has demonstrated that IL-6 plays a critical role in cancer progression and resistance to therapeutics. Therefore, the effect of IL-6 on breast cancer cells has been inconsistent (20).

On the one hand, IL-6 displays antitumorigenic effects on cancer cells. Several studies have reported that IL-6 inhibits proliferation and induces apoptosis in MCF-7 cells (21). The possible mechanism may activate the immune cell response. Some studies have demonstrated that endotoxin induces immune cell recruitment and activation, and proinflammatory cytokines have been considered anticarcinogenic agents (22). Moreover, 4T1 cell proliferation and migration were inhibited when cells were cultured in the conditioned medium of RAW 264.7 cells stimulated by LPS, indicating that inflammatory mediators promoted anticancer properties (23). In this study, raising IL-6 production may promote chemoprevention, consistent with other studies.

On the other hand, IL-6 acts opposing protumorigenic cytokines through a STAT3-mediated process (24). IL-6 signaling is closely related to MDA-MB 231 cell growth and *in vivo* malignancy; blocking its pathway was associated with inhibition of tumor growth and invasion (25). Higher serum IL-6 levels in breast cancer patients correlate with clinical stage, poor prognosis, and a low survival rate (26). This opposing switch shares signaling through a receptor combination explanted for protumorigenic and antitumorigenic activities (24). Possible signaling through IL-6 receptors divides into a membrane form (mIL-6R), as a classic signaling response to anti-inflammation, and a soluble form (sIL-6R), as a trans signal response to proinflammation (27). Therefore, IL-6/IL-6R is a target for cancer therapies by using antagonists, monoclonal antibodies, or shRNA, suppressing cancer stem cells, colony formation, and tumor development (24).

Another mediator, PGE_2_, is the major derivative of cyclooxygenase (COX) catalysis and has been widely discussed as a hallmark of the link between inflammation and tumor development by promoting proliferation, invasion, metastasis, and angiogenesis (28). Accordingly, non-steroidal anti-inflammatory drugs, such as aspirin, act to inhibit PGE_2_ production and have been demonstrated to inhibit tumor initiation and progression (29, 30). It has been revealed that the overexpression of COX-2 is a prominent characteristic in all clinical stages of breast cancer patients (29). Proinflammatory cytokines such as IL-6 may participate in COX-2 gene promoter binding sites and trigger signal transduction cascades, enhancing PGE_2_ biosynthesis (29). Therefore, in MCF-7 cells, lunasin-induced COX-2 mRNA and PGE_2_ secretion might indicate the induction of IL-6 production at 24 h. In MDA-MB-231 cells, lunasin decreased COX-2 mRNA expression but did not affect PGE_2_ secretion, indicating that this effect is not dependent on IL-6 regulation. Accordingly, lunasin regulated inflammatory mediators through various pathways in different breast cancer cells.

In breast cancer, leptin and leptin receptor (ObR) have been reported to be associated with distant metastasis, worse prognosis, and poor survival (31). ObR has been found in breast cancer MCF-7, MDA-MB-231, and HCC 1937 cells, and leptin supplementation stimulated cell proliferation (32). The possible mechanisms have revealed that leptin participates in fundamental signaling pathways of adhesion, angiogenesis, migration, inflammation, and proliferation via the Wnt/β-catenin, JAK-STAT, PI3K, and mitogen-activated protein kinases (MAPK) pathways (31, 32). Moreover, NLRP3 inflammasome activation by leptin supplementation induced breast cancer growth (33). The expression of ObR in breast cancer cells is a survival mechanism that defenses chemotherapy damage (32). Therefore, the critical role of leptin in pathogenesis provides potential therapeutic strategies for breast cancer. In this study, lunasin suppressed Ob-R gene expression in MCF-7 cells but not in other cells, suggesting that this suppression might contribute to the chemoprevention of MCF-7 cells.

VEGF is one of the critical angiogenic factors in breast cancer cell proliferation, vessel formation, migration, and endothelial cell remodeling in supporting tumor progression. Typically, the expression of VEGF is higher in TNBC than in other types of breast cancer, and this increased level is also related to poor outcomes (34). Therefore, using antiangiogenic factors to inhibit vasculature might control cell proliferation and was proposed for cancer therapy (35). In 4T1 cells, the VEGF level was positively correlated with cell viability and migration (36). In this study, lunasin inhibited VEGF secretion in both cancer cell lines, contributing to the chemoprevention of breast cancer.

In addition to inflammatory mediators, hormone signaling acts on physiologic functions either locally or in systematic circulation and affects breast cancer development (5). Estrogen is mainly present as 17β-estradiol in humans; other types of estrogen were found to be estron and estriol. Scientists have revealed that estrogen is one of the risk factors for worsening breast cancer. In premenopausal women, estrogen is secreted from the ovary; nevertheless, in postmenopausal women, the primary source of estrogen is secreted from adipocytes by aromatase catalysis, which is positively correlated with an increased risk of breast cancer (4, 17). Aromatase, one of the cytochrome P450 superfamily members, transfers androstenedione and testosterone to estron and estradiol, displaying the physical form of estrogen *in vivo* (6). As research has shown, estrogens influence the etiology of ER+ and ER− breast tumors, leading to metastasis, invasion, and migration (17). Therefore, aromatase inhibitors have been considered one of the target therapies for both ER+ and ER− breast cancer cells (37, 38).

In the present study, the β-estradiol condition only increased the cell growth of MCF-7 cells; additionally, lunasin treatment still suppressed cell vitality and growth. Consistent with another laboratory, MCF-7 cells cultured in the presence of β-estradiol had a shortened cell cycle duration (39). Estrogen signaling is mediated via its specific receptor on target cells, including ERα and ERβ, when it binds to estrogen and interacts with its responsive element on the promoters of target genes. ERα signaling plays a predominantly immunostimulatory role in neoplastic transformation and cell proliferation, while ERβ activation appears to have an immunosuppressive effect and a contradictory effect of ERα in breast cancers (17). This result indicated that compounds that exhibit estrogenic activity toward ERα or ERβ might affect the course of diseases. Therefore, selective target-ERα signaling would be the desired compound in pharmaceutical strategies to cure breast cancer (17).

An early study reported that MDA-MB-231 cells express aromatase and sulfatase, which are required for β-estradiol synthesis. Moreover, cells express low ERβ and no ERα, suggesting that these cells still respond to estrogens through non-ER-mediated pathways (38, 40). One laboratory used ERβ agonists to treat MDA-MB-231 cells and showed decreased cell growth and invasion, suggesting that ERβ may suppress invasion in an ERα-independent manner (41). Furthermore, aromatase induces estrogen formation, which affects breast cell growth; thus, aromatase inhibitors remarkably increase chemopreventive efficacy compared to the current therapeutic options, and they are also considered safer. In this study, MDA-MB-231 cells expressed lower ERα, ERβ, and aromatase gene levels than MCF-7 cells. Interestingly, lunasin not only inhibited aromatase gene expression and activity but also decreased ERα gene expression. Additionally, lunasin apparently increased ERβ gene levels, especially in MDA-MB-231 cells, suggesting that the regulation of estrogenic molecules might contribute to the chemopreventive properties of lunasin.

Moreover, proinflammatory mediators have been proposed to play critical roles closely linked to estrogen production and signaling (17). Inflammatory factors, such as PGE_2_ and IL-6 production, increase aromatase activity, accelerate estrogen production, and deteriorate breast cancer (17, 26). In both breast cancer cell lines in this study, lunasin regulated ERα/β genes and the aromatase gene and its activity at 24 h, indicating that this suppressive property might not be dependent on the IL-6 pathway. At the moment, lunasin first induced IL-6 production immediately to suppress cancer cell growth and subsequently decreased its secretion to maintain homeostasis, suggesting that lunasin regulated inflammation in this microenvironment.

Instead of traditional therapy with side effects, natural compounds could be the most intriguing aspect of chemopreventive intervention on cancer progression (42). In the past decade, laboratory data have demonstrated that lunasin is a probable chemopreventive agent in breast cancer (9). Lunasin exerted a synergistic effect in breast cancer chemoprevention by arresting the cell cycle and inducing apoptosis (12). In a xenograft model of MDA-MB-231 cells, lunasin was found to lower tumor incidence, weight, and malignant histology in mice (10). The possible mechanism of action has been revealed to exert epigenetic action by inhibiting core histone H3 and H4 acetylation and proteins of cyclins and cyclin-dependent kinases (13). In both MCF-7 and MDA-MB-231 cells, lunasin inhibited cell migration and invasion by downregulating integrin signaling through FAK/Akt/ERK and NF-κB and matrix metalloproteinases-2/-9 (43). Additionally, the antimetastatic activity of lunasin in 4T1 TNBCs has also been proven by the suppression of the angiogenetic mediator VEGF (14). Based on this evidence, lunasin acts as a promising chemopreventive agent for both ER+ and ER− breast cancer cells by arresting the cell cycle, inducing apoptosis, regulating epigenesis, and suppressing integrin signaling and angiogenesis mediators.

This study first proposed that lunasin regulated inflammatory mediators and estrogenic molecules and decreased aromatase activity, resulting in the induction of apoptosis and the inhibition of cell growth in both MDA-MB-231 and MCF-7 cells. In summary, lunasin regulates inflammatory mediators and estrogen-related molecules, providing novel insights into effective therapeutic strategies for breast cancer. Further studies could be carried out on lunasin’s performance in animal and clinical studies, and its effective dosage might benefit future cancer therapies.

## Supplementary Material

Seed-derived peptide lunasin suppressed breast cancer cell growth by regulating inflammatory mediators, aromatase, and estrogen receptors

## Supporting information

Supplementary 1. The cycle number of gene expression in breast cancer cells treated with lunasin by qPCR assay. Supplementary 2. Inflammatory cytokine secretion in breast cancer cells treated with lunasin by ELISA assay.
