# Supplementary material for: Seed-derived peptide lunasin suppressed breast cancer cell growth by regulating inflammatory mediators, aromatase, and estrogen receptors
Source: Food Nutr Res. 2023 Jan 26;67:10.29219/fnr.v67.8991. doi: 10.29219/fnr.v67.8991 (PMC9899045; doi:10.29219/fnr.v67.8991)
Supplement: Seed-derived peptide lunasin suppressed breast cancer cell growth by regulating inflammatory mediators, aromatase, and estrogen receptors [file FNR-67-8991-s001.docx]

**Supplementary 1**. The cycle number of genes expression in breast cancer cells treated with lunasin by qPCR assay ^a^

| MDA-MB-231 cell | | | | | | | | |
| --- | --- | --- | --- | --- | --- | --- | --- | --- |
| Group | GAPDH | Erα | Erβ | Aromatase | COX-2 | HIF-1α | Ob-R | IL-6 |
| 0 | 18.5 ± 0.2 | 28.3 ± 0.3 | 30.4 ± 0.3 | 29.9 ± 0.6 | 28.7 ± 0.9 | 26.9 ± 1.7 | 28.3 ± 0.8 | 21.3 ± 0.7 |
| Lun 5μM | 18.6 ± 0.4 | 29.1 ± 0.2 | 29.5 ± 0.5 | 29.8 ± 0.2 | 28.9 ± 1.7 | 27.4 ± 2.4 | 27.9 ± 1.3 | 21.2 ± 1.5 |
| Lun 50μM | 17.8 ± 0.4 | 29.9 ± 1.1 | 29.1 ± 0.3 | 31.1 ± 2.0 | 28.3 ± 1.4 | 26.5 ± 2.0 | 27.4 ± 2.0 | 20.2 ± 0.4 |
| MCF-7 cell | | | | | | | | |
| Group | GAPDH | Erα | Erβ | Aromatase | COX-2 | HIF-1α | Ob-R | IL-6 |
| 0 | 16.2 ± 1.0 | 17.6 ± 0.9 | 19.1 ± 1.6 | 24.1 ± 1.1 | 23.4 ± 5.8 | 28.4 ± 0.7 | 20.2 ± 4.0 | 22.0 ± 2.9 |
| Lun 5μM | 16.1 ± 0.7 | 18.0 ± 0.8 | 18.8 ± 1.2 | 24.8 ± 1.3 | 23.2 ± 5.2 | 28.5 ± 1.0 | 20.3 ± 3.0 | 21.5 ± 2.1 |
| Lun 50μM | 16.8 ± 0.9 | 19.2 ± 1.3 | 19.1 ± 1.4 | 25.9 ± 1.6 | 24.1 ± 4.9 | 29.2 ± 2.5 | 22.1 ± 3.2 | - 1. ± 2.7 |

^a.^MDA-MB-231 and MCF-7 cells treated with lunasin for 24 h, and the cycle number of analysis in qPCR assay. The value of the data is presented as mean ± SEM.

**Supplementary 2.** Inflammatory and angiogenic cytokines secretion in breast cancer cells treated with lunasin by ELISA assay ^ab^

|  | MDA-MB-231 cell | | | | |
| --- | --- | --- | --- | --- | --- |
| Group |  | PGE_2_ (pg/mL) | IL-6 (pg/mL) | VEGF  (pg/mL) | IL-1β (pg/mL) |
| 24h | 0 | 55.4 ± 2.4 | 1019 ± 4.1 | 623 ± 7.6 | 3.0 ± 0.1 |
|  | Lun 5 μM | 50.6 ± 2.4 | 1037 ± 5.6* | 523 ± 8.3* | 3.0 ± 0.1 |
|  | Lun 50 μM | 53.1 ± 6.2 | 1098 ± 5.7* | 574 ± 8.8* | 3.1 ± 0.1 |
| 48h | 0 | 45.3 ± 2.0 | 1021 ± 9.1 | 580 ± 8.3 | 17.0 ± 0.3 |
|  | Lun 5 μM | 42.7 ± 3.4 | 949 ± 8.1* | 520 ± 12.4* | 16.7 ± 0.4 |
|  | Lun 50 μM | 40.9 ± 2.2* | 1041 ± 8.5 | 579 ± 7.6 | 16.4 ± 0.4 |
|  |  |  | MCF-7 cell | | |
| Group |  | PGE_2_ (pg/mL) | IL-6 (pg/mL) | VEGF  (pg/mL) | IL-1β (pg/mL) |
| 24h | 0 | 25.4 ± 0.8 | 36.8 ± 2.5 | 180 ± 10.8 | 1.6 ± 0.2 |
|  | Lun 5 μM | 32.6 ± 1.1* | 50.3 ± 2.0* | 195 ± 3.9 | 1.9 ± 0.1 |
|  | Lun 50 μM | 29.6 ± 3.0 | 53.5 ± 1.3* | 199 ± 9.1 | 2.0 ± 0.3 |
| 48h | 0 | 19.1 ± 2.8 | 98.1 ± 1.3 | 323 ± 4.8 | 7.6 ± 0.6 |
|  | Lun 5 μM | 22.6 ± 3.2 | 97.4 ± 1.7 | 271 ± 9.1* | 2.9 ± 0.2* |
|  | Lun 50 μM | 17.3 ± 1.3 | 63.0 ± 3.3* | 320 ± 6.5 | 2.8 ± 0.2* |

^a.^ MDA-MB-231, and MCF-7 cells treated with lunasin for 24 h and 48 h, and culture supernatants were collected for cytokines analysis by ELISA.

^b.^ The value of data is presented as mean ± SEM, and statistical analysis was done by independent sample t-test; significant differences of control displayed in * *p* < 0.05.
